# Antifungal Potential of Marine Organisms of the Yucatan Peninsula (Mexico) against Medically Important *Candida* spp.

**DOI:** 10.3390/molecules28020606

**Published:** 2023-01-06

**Authors:** Dawrin Pech-Puch, Diana Grilo, Susana Eunice Calva-Pérez, Andreia Pedras, Harold Villegas-Hernández, Sergio Guillén-Hernández, Raúl Díaz-Gamboa, Mateo Forero Tunjano, Jaime Rodríguez, Oscar A. Lenis-Rojas, Carlos Jiménez, Catarina Pimentel

**Affiliations:** 1 Departamento de Biología Marina, Universidad Autónoma de Yucatán, Km. 15.5, Carretera Mérida-Xmatkuil, A.P. 4-116 Itzimná, Merida CP 97100, Mexico; 2 Escuela Nacional de Estudios Superiores Unidad Mérida (ENES Mérida), Universidad Nacional Autónoma de México (UNAM), Carretera Mérida-Tetiz km 4.5, Tablaje, Catastral No. 6998, Municipio de Ucú, Ucú CP 97357, Mexico; 3 Instituto de Tecnologia Química e Biológica António Xavier (ITQB NOVA), Universidade Nova de Lisboa, Oeiras, 1070-312 Lisbon, Portugal; 4 Centro de Interdisciplinar de Química e Bioloxía (CICA), Facultade de Ciencias, Universidade da Coruña, 15071 Coruña, Spain

**Keywords:** marine natural products, Yucatan Peninsula, antifungal, *Candida*

## Abstract

Invasive fungal infections represent a global health threat. They are associated with high mortality and morbidity rates, partly due to the ineffectiveness of the available antifungal agents. The rampant increase in infections recalcitrant to the current antifungals has worsened this scenario and made the discovery of new and more effective antifungals a pressing health issue. In this study, 65 extracts from marine organisms of the Yucatan Peninsula, Mexico, were screened for antifungal activity against *Candida albicans* and *Candida glabrata*, two of the most prevalent fungal species that cause nosocomial invasive fungal infections worldwide. A total of 51 sponges, 13 ascidians and 1 gorgonian were collected from the coral reef and mangrove forest in the Yucatan Peninsula (Mexico) and extracted with organic solvents. Nine crude extracts showed potent antifungal activity, of which four extracts from the sponge species *Aiolochroia crassa*, *Amphimedon compressa*, *Monanchora arbuscula* and *Agelas citrina* had promising activity against *Candida* spp. Bioassay-guided fractionation of the *M. arbuscula* extract revealed the remarkable fungicidal activity of some fractions. Analysis of the chemical composition of one of the most active fractions by UHPLC-HRMS and NMR indicated the presence of mirabilin B and penaresidin B, and their contribution to the observed antifungal activity is discussed. Overall, this work highlights marine organisms of the Yucatan Peninsula as important reservoirs of natural products with promising fungicidal activity, which may greatly advance the treatment of invasive fungal infections, especially those afflicting immunosuppressed patients.

## 1. Introduction

Fungi are important components of most ecosystems on Earth [1]. They are also part of the human microbiota and, under particular circumstances, can cause health-threatening invasive infections, in which the fungus reaches the bloodstream or any major internal organs [2]. The yeast *Candida* spp. asymptomatically colonizes the skin, mucosal surfaces and the gastrointestinal tract of most healthy individuals [3]. However, some aggressive drug therapies or immunosuppressive infections may promote their transition from commensals to pathogens [4]. As a result, invasive fungal infections caused by commensal *Candida* spp. are the most prevalent severe fungal infections among hospitalized patients [4,5]. The mortality rates of patients with invasive candidiasis are unacceptably high, even for those who receive timely antifungal therapy [6]. The disease is also associated with high healthcare costs and prolonged hospital stays [7].

*Candida albicans* is the most frequent causative agent of nosocomial invasive fungal infections [5], followed by *Candida glabrata* and *Candida parapsilosis* [8]. *C. glabrata* has recently emerged as an important pathogen, due to its inherent tolerance to the most prescribed antifungal worldwide—fluconazole—and the rampant increase in clinical isolates that are resistant to the available drugs [9].

**Figure 1:**
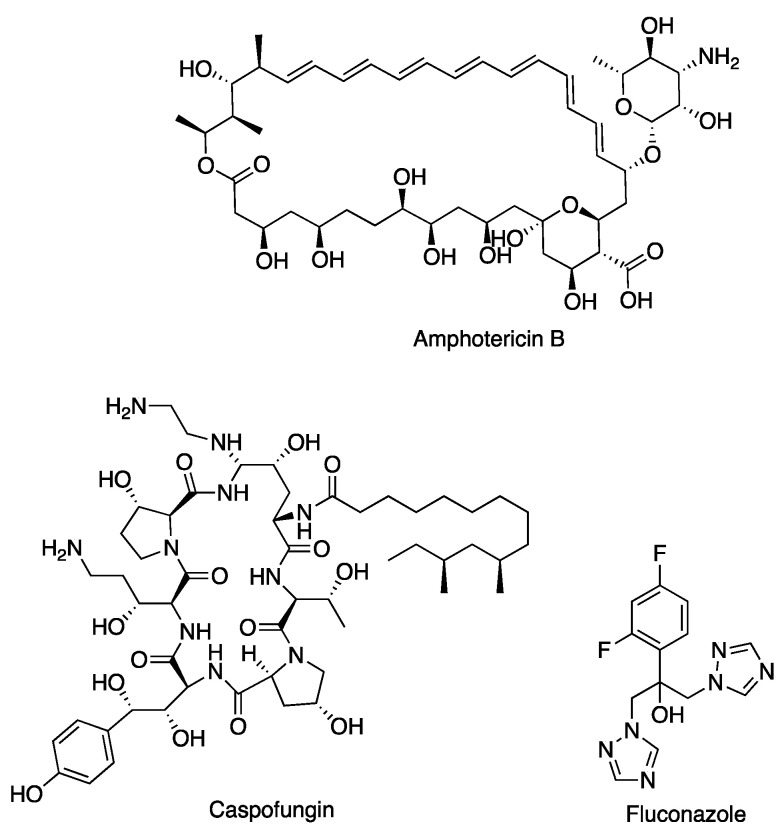
Chemical structure of representatives of the three classes of antifungal drugs used in the treatment of invasive fungal infections: amphotericin B (polyene), caspofungin (echinocandin), and fluconazole (azole).

Only the following three primary classes of antifungal agents are currently available to treat invasive fungal infections: polyenes, azoles, and echinocandins [10] (Figure 1). The recent emergence of fungi resistant to one or more classes of antifungals has compromised the effectiveness of treatment, and significantly reduced the repertoire of antifungal agents that clinicians have to fight serious fungal infections [11]. This concerning situation has led the WHO (World Health Organization) and CDC (Centers for Disease Control and Prevention) to conclude that tackling *Candida* spp. antifungal resistance should be considered a priority [12]. Clearly, there is an urgent need to develop alternative antifungals that are capable of bypassing the known mechanisms of drug resistance.

The large size and biological diversity of the oceans make them promising natural sources of bioactive molecules [13]. In the marine environment, organisms subjected to a plethora of stimuli produce molecules with unique structural, chemical, and biological characteristics. These marine natural products (MNPs) are a rich source of potential alternative drugs [14], many of which have already entered clinical phase trials [13].

Mexico has been considered as one of the three areas in the world with the greatest terrestrial and marine biodiversity. In particular, the Gulf of Mexico and the Caribbean Sea, which meet in the Yucatan channel, constitute two outstanding marine ecosystems [15]. The single geographical location of the Yucatan channel promotes the abundance of highly diverse and unique marine species, which represent a potential source of bioactive compounds [15,16,17,18].

The antifungal potential of the marine organisms of the Yucatan Peninsula (YP) has not been intensively investigated [15]. The macroalgae are the only group of marine organisms in the YP that have been searched for antifungal activity. Indeed, as far as we know, there are only two reports on this topic. The first, by Morales et al. [19], evaluated the antifungal activity present in the extracts of marine macroalgae against *Trichophyton mentagrophytes.* In the second, the antifungal activity of marine macroalgae extracts on *Pseudocercospora fijiensis*, *Colletotrichum gloeosporioides* and *Fusarium oxysporum* was tested [20].

In this work, we report the promising antifungal activity of 65 extracts from several marine invertebrate species of the YP against yeast species that cause life-threatening infections.

## 2. Results and Discussion

### 2.1. Screening of a Library of Marine Extracts from the Yucatan Peninsula for Antifungal Activity

A library of 65 extracts (51 sponges, 13 ascidians and 1 gorgonian), collected from the coral reef and mangrove forest in the Yucatan Peninsula in Mexico, was screened for antifungal activity against *C. albicans* and *C. glabrata*. The crude extracts were resuspended in the smallest possible volume of DMSO, yielding the stock concentrations listed in Table 1. To ensure total solubilization, all the extracts were sonicated prior to use. Next, 5 µL of each extract (Table 1) was added to the wells of a 96-well plate, containing a cellular suspension of *C. glabrata* or *C. albicans*. Growth was recorded after 48 h at 30 °C (*C. albicans*) or 37 °C (*C. glabrata*), by measuring OD_600_. Growth ratios were determined in comparison to the control cells and those below 0.5 were considered to be active extracts.

**Figure 2:**
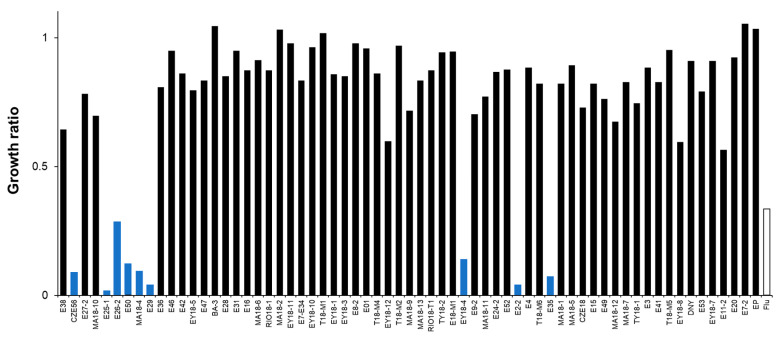
Susceptibility of *C. glabrata* to marine extracts from the Yucatan Peninsula. Growth was monitored after 48 h of incubation at 37 °C. Growth ratios were determined in comparison to control (untreated) cells. Fluconazole (a triazole antifungal) was used as a reference antifungal (16 µg/mL). Growth ratios below 0.5 indicate active extracts (blue bars).

**Figure 3:**
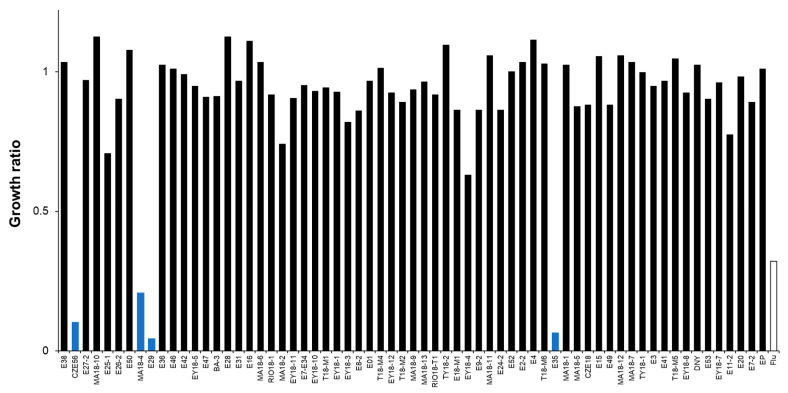
Susceptibility of *C. albicans* to marine extracts from the Yucatan Peninsula. Growth was monitored after 48 h of incubation at 30 °C. Growth ratios were determined in comparison to control (untreated) cells. Fluconazole (a triazole antifungal) was used as a reference antifungal (0.5 µg/mL). Growth ratios below 0.5 indicate active extracts (blue bars).

For the concentrations tested (Table 1), 9 of the 65 extracts, obtained from 8 sponge species, showed antifungal activity against *C. glabrata* (Figure 2). The active extracts were from *Aiolochroia crassa* (collected from two different locations: Mahahual in the Quintana Roo state (MA18-4) and Alacranes Reef in the Yucatan state (E50)), *Amphimedon compressa*, *Monanchora arbuscula*, *Leucetta floridana*, *Agelas sceptrum*, *A. citrina*, *A. dilatata*, and *Haliclona (Rhizoniera) curacaoensis*. Four of these extracts (*A. crassa* (MA18-4), *A. compressa* (E29), *M. arbuscula* (E35) and *A. citrina* (CZE56)) also had antifungal activity against *C. albicans* (Figure 3). To the best of our knowledge, there are no previous studies on the antifungal activity of the following three sponges: *H. (Rhizoniera) curacaoensis, A. crassa*, and *A. dilatata*.

The antifungal activity displayed by the *A. citrina* extract may be due to the presence of agelasidines (Table 10, Figure 10). It has been reported that the alkaloid (–)-agelasidine C shows strong antifungal activity on *C. albicans* [21]. Moreover, (–)-agelasidine C and agelasidines E and F, isolated from *A. citrina*, also showed activity against *C. albicans* [21,22].

As for *A. compressa*, methanol extracts obtained from this species have already demonstrated antifungal activity against *C. albicans* [23]. Accordingly, 8,8′-dienecyclostellettamine (Table 1) isolated from this species is active against *C. glabrata* and *C. albicans* [24].

Organic extracts from *A. sceptrum* (E26-2) were also strongly active against *C. glabrata*, but less active against *C. albicans* (Figure 2 and Figure 3, respectively). A compound with antifungal activity against *C. albicans* and *Alternaria* sp. known as sceptrin was previously isolated from this species [25].

*L. floridana*-derived extracts are known to display antifungal activity on *C. albicans* [26]. In this study, we found that *L. floridana* crude extracts are very active against *C. glabrata* (Figure 2).

Batzelladine L, batzelladine D, norbatzelladine L, and ptilomycalin A were isolated from *M. arbuscula* with high antifungal activity against clinically important fungi. Batzelladin L is active against the filamentous fungus *Aspergillus flavus* [27], batzelladines D and norbatzelladine L are active against *Saccharomyces cerevisiae*, a yeast that is phylogenetically close to *C. glabrata* [28], and the alkaloid ptilomycalin A is active against the yeast *Cryptococcus neoformans* [29].

We determined the minimum inhibitory concentration (MIC) of the four most active extracts against both *Candida* spp. (Table 2). The extracts were serial diluted in DMSO and 5 µL was added to the wells of a 96-well plate, containing cellular suspensions of *C. glabrata* or *C. albicans*. Growth was recorded after 24 and 48 h at 30 °C (*C. albicans*) or 37 °C (*C. glabrata*), by measuring OD_600_. The MIC was defined as the drug concentration where the relative OD_600_ fell at least 50% below the control (DMSO alone).

*M. arbuscula* (E35) was the most active extract against both *Candida* spp. with an MIC of 3.91 μg/mL (Table 2). The extract of *A. compressa* was more active against *C. glabrata* than against *C. albicans* and the opposite was observed for the extract of *A. citrina*. The *A. crassa* extract was the least active against both species. The MIC for *A. compressa*, *M. arbuscula*, and *A. citrina* did not change between 24 and 48 h.

The potent activity of the crude extract from *M. arbuscula* (E35) on *C. albicans* and *C. glabrata* led us to further explore this extract.

### 2.2. Bioassay-Guided Fractionation of the M. arbuscula Extract

**Figure 4:**
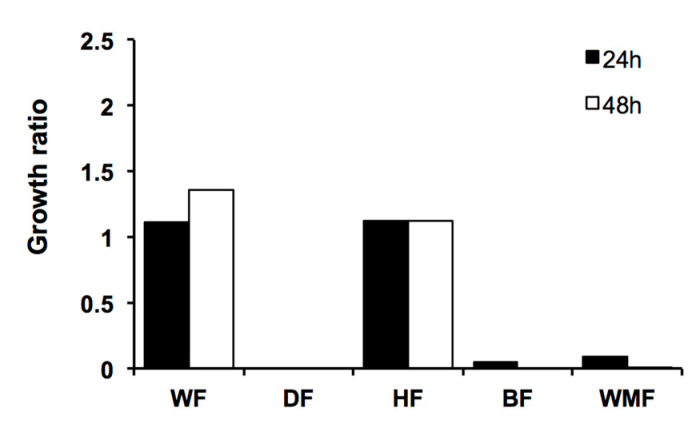
Susceptibility of *C. glabrata* to *M. arbuscula* fractions. Growth was monitored after 48 h of incubation at 37 °C. Growth ratios were determined in comparison to control (untreated) cells. Growth ratios below 0.5 indicate active fractions.

**Figure 5:**
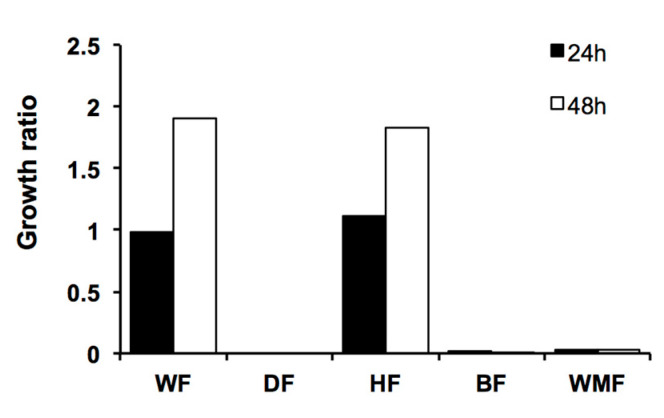
Susceptibility of *C. albicans* to *M. arbuscula* fractions. Growth was monitored after 48 h of incubation at 30 °C. Growth ratios were determined in comparison to control (untreated) cells. Growth ratios below 0.5 indicate active fractions.

The *M. arbuscula* crude extract was partitioned using the modified Kupchan procedure to obtain the following five fractions: E35-WF, E35-BF, E35-HF, E35-WMF, and E35-DF (Table 3). The three most active fractions against both *C. glabrata* and *C. albicans* were E35-DF, E35-BF, and E35-WMF (Table 3, Figure 4 and Figure 5), with E35-DF being the most active fraction.

The fraction E35-DF, which required the slightest amount to produce a significant impact on yeast growth (Table 3), was then subjected to solid phase extraction (SPE) using an RP-18 cartridge. The procedure generated seven sub-fractions, R1–R7 (Table 4), whose fungistatic and fungicidal activity were evaluated on *C. glabrata* and *C. albicans*.

**Figure 6:**
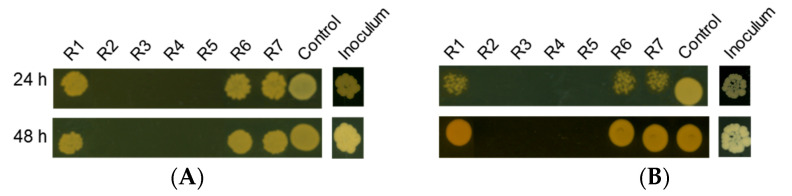
Growth of *Candida* spp. with different sub-fractions of E35-DF. Yeast cells were incubated with the sub-fractions for 24 h and 48 h and a volume of 5 μL was spotted onto YPD agar plates. Images were digitalized after 24 h of incubation at 37 °C for *C. glabrata* (**A**) or 30 °C for *C. albicans* (**B**). The concentrations tested are listed in Table 4. Inoculum: growth prior to sub-fraction addition; untreated control cells.

We found that all the sub-fractions were active against *C. glabrata* and *C. albicans,* with the sub-fractions R2, R3, R4 and R5 exceling in terms of antifungal efficacy at the concentrations assayed (Figure 6 and Table 5).

The MICs of the most active sub-fractions (R2 to R5) were determined next (Table 5). With the exception of sub-fraction R4, all the other sub-fractions had lower MICs than the original E35-FD fraction (Table 5), confirming the success of the fractionation step.

In addition to *C. albicans* and *C. glabrata,* other *Candida* spp. are emerging as important pathogens. Among them are *C. krusei, C. tropicalis*, and *C. parapsilosis,* which together with the former are responsible for more than 90 percent of all yeast infections [30]. Therefore, the most active fractions R2–R5 were also tested against *C*. *krusei*, *C*. *tropicalis*, and *C*. *parapsilosis* (Table 6).

All of the sub-fractions showed activity against *C. krusei, C. tropicalis,* and *C. parapsilosis*, with sub-fraction R4 being the most active one.

By determining the minimum fungicidal concentration (MFC), we also found that sub-fractions R4 and R5 generally had the highest fungicidal activity (lower MFCs) against all species at 24 and 48 h (at concentrations that were two to eight times higher than the MIC). The exception was *C. parapsilosis*, for which the sub-fractions R2 and R3 had the strongest activity at 48 h (Table 7).

### 2.3. De-Replication Analysis of the Sub-Fractions from M. arbuscula

**Figure 7:**
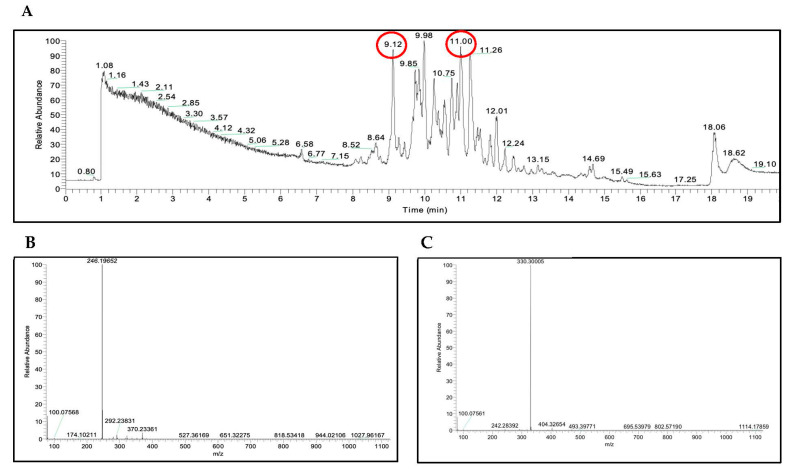
UHPLC-HRMS analysis of sub-fraction R4. (**A**) UHPLC chromatogram. Peaks analyzed by HRMS are marked with a red circle. (**B**) HRMS of the chromatographic peak from the sub-fraction R4 eluted with a retention time of 9.12, showing an [M + H]^+^ ion adduct that matches the molecular formula of C_15_H_23_N_3_ for mirabilin B. (**C**) HRMS of the chromatographic peak from the sub-fraction R4 eluted with a retention time of 11.0 with an [M + H]^+^ ion adduct that matches the molecular formula of C_19_H_39_NO_3_ for penaresidin B.

As a first attempt to identify the compounds responsible for the promising antifungal activity of sub-fractions R2, R3, R4 and R5, de-replication analyses of these fractions were carried out using the UHPLC-HRMS positive mode (see Supplementary Materials for R2, R3, and R5 UHPLC-HRMS experiment; Figures S1–S3 and Figure 7 for R4). The [M + H]^+^ ion adducts that corresponded to all the signals detected in the LC/MS chromatograms were analyzed using the Antimarin^®^ and Scifinder^®^ platforms.

A total of 19 [M + H]^+^ ion adducts that corresponded to 19 UHPLC signals were detected, including 3 for R2, 2 for R3, 7 for R4, and 7 for R5 (Table 8).

**Figure 8:**
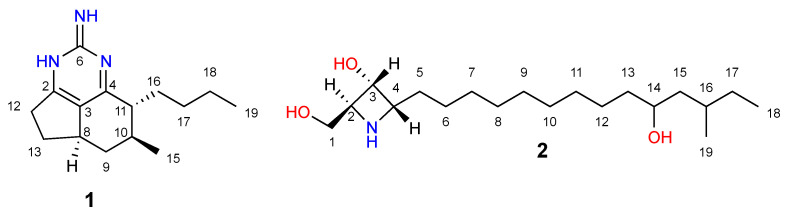
Chemical structure of compounds mirabilin B (**1**) and penaresidin B (**2**).

Two compounds that corresponded to the [M + H]^+^ ion adducts found in the R4 sub-fraction were identified as mirabilin B and penaresidin B (Figure 8). The HRMS of the compounds eluted with a retention time of 9.12 min showed an [M + H]^+^ ion adduct at *m/z* 246.1965, matching that of mirabilin B [33] (calculated as *m*/*z* 246.1964, Figure 7B). The other match, with a retention time of 11.00 min and an experimental value of [M + H]^+^ *m/z* 330.3001, corresponded to penaresidin B [31,32] (calculated as *m/z* 330.3002, Figure 7C, Figures S5 and S7). The remaining [M + H]^+^ ion adducts could not be identified; therefore, they may be either compounds not yet included in the Antimarin^®^ and Scifinder^®^ platforms or new compounds.

To confirm the presence of mirabilin B (**1**) and penaresidin B (**2**) (Figure 8) in the R4 sub-fraction, the carbon chemical shift signals of the ^13^C NMR spectrum of this sub-fraction (see Supplementary Materials, Figures S4 and S6) were compared to those reported for these compounds in the literature, using a Pearson’s chi-squared goodness of fit test ($\chi^{2}$) with Yates continuity correction (Table 9). The ^13^C NMR spectrum of the R4 sub-fraction indicated the presence of the main carbon chemical shifts of compounds **1** and **2**.

The chi-squared goodness of fit test revealed that the experimental values for compound (**1**) did not differ significantly, at a 99% confidence level, from the reported values ($\chi_{Y}^{2}=0.0460$; *p*-value = 0.8302). The same was observed for compound (**2**) ($\chi_{Y}^{2}=0.0405$; *p*-value = 0.8405). Thus, we conclude that the experimental data do not differ significantly from those expected for both compounds.

Mirabilin B was identified in *M. arbuscula* (previously known as *Monanchora unguifera*) and demonstrated activity against *Cryptococcus neoformans* [34], but there are no data on its activity against *Candida* spp. Penaresidin B, isolated from the marine sponge *Penares* sp., has no antifungal activity against *C. neoformans*, *Aspergillus niger*, or *C. albicans* [35]. Although at this stage, we cannot rule out the contribution of the other compounds (Table 8), it may well be that the antifungal activity observed in R4 results from mirabilin B or from its synergetic interaction with those unidentified NPs.

## 3. Materials and Methods

### 3.1. General Experimental Procedures

The separation was performed using a Waters XBridge column C18, 2.1 × 150 mm, 3.5 µm particle size, P/N 186003023 (Optima™ LC/MS Grade, Thermo Fisher Scientific, Waltham, MA, USA). The column temperature was maintained at 30 °C. The data were acquired on Q Exactive Focus (Thermo Fisher Scientific, Waltham, MA, USA) coupled to UHPLC, using Xcalibur software v.4.0.27.19 (Thermo Fisher Scientific, Waltham, MA, USA). The method consisted of several cycles of full MS scans (R = 70,000) in positive mode and negative mode in separate runs. External calibration was performed using the LTQ VELOS ESI Positive Ion Calibration Solution (ref.: 11340360, Thermo Fisher Scientific, Waltham, MA, USA) and Negative Ion Calibration Solution (ref.: 11360360, Thermo Fisher Scientific, Waltham, MA, USA). The raw MS was analyzed using Compound Discoverer software v2.1 (Thermo Fisher Scientific, Waltham, MA, USA).

^13^C NMR spectra were recorded on a Bruker Avance 500 spectrometer at 125 MHz, using CDCl_3_. The chromatographic analysis was performed on an UltiMate 3000 UHPLC (Thermo Fisher Scientific, Waltham, MA, USA).

### 3.2. Statistical Analyses

A Pearson’s chi-squared goodness of fit test ($\chi^{2}$) was applied to determine whether our data (experimental chemical shift values of ^13^C NMR for compound (**1**)) were significantly different from those expected (reported chemical shift values of ^13^C NMR for compound **1**). The same procedure was carried out for compound (**2**). It is worth mentioning that both $\chi^{2}$ tests were applied with the Yates continuity correction to reduce the approximation error, and thus prevent overestimation of the statistical significance for small data [36]. Herein, the chi-squared statistic was as follows:

$$\chi_{Y}^{2}=\overset{n}{\underset{i=1}{\sum }}\frac{{\left(\left|O_{i}-E_{i}\right|-0.5\right)}^{2}}{E_{i}}$$

where $O_{i}$ would represent the observed values, and $E_{i}$ would be the expected values.

### 3.3. Animal Collection and Identification

**Figure 9:**
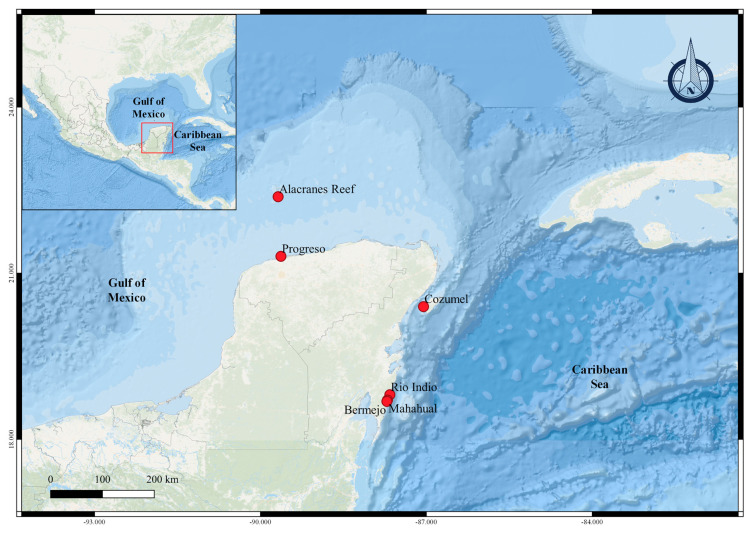
Collection sites of marine organisms in the Yucatan Peninsula, Mexico.

Samples of animals were collected by snorkeling and scuba diving in different coastal zones of the Yucatan Peninsula, Mexico, during the following three different periods: September–December 2016, January–March 2017, and September 2018. The selected species were collected from the following two different regions: Mexican Caribbean (Cozumel Island, Rio Indio, Mahahual, and Bermejo, Quintana Roo) and Campeche Bank (Alacranes Reef and Progreso, Yucatan) (Figure 9).

**Figure 10:**
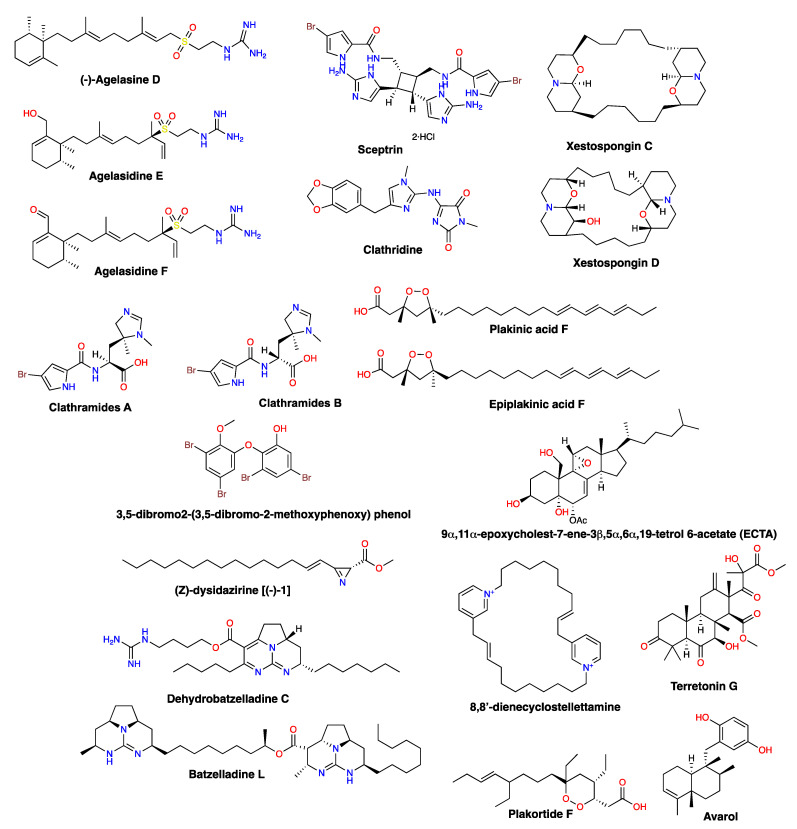
Selected structures of compounds with reported antifungal activity isolated from marine organisms.

The sponges were identified at the ICMyL-UNAM (Mexico), while the ascidians were identified at the University of Vigo (Spain) and Autonomous University of Yucatan (Mexico). Taxonomic information, collection sites, and previous reports on the antifungal activity of the species/genus of the 65 marine organisms are shown in Table 10. The structures of the compounds with antifungal activity previously isolated from marine species, whose extracts were tested in this work, are depicted in Figure 10.

### 3.4. Preparation of the Organic Extracts

Tissue slices of each species were exhaustively extracted three times in a lapse of 24 h each, with a 500 mL mixture of dichloromethane–methanol (1:1), at 25 °C. The solvent was filtered and then removed under vacuum at 40 °C with a rotatory evaporator. The extracts were stored at −20 °C in tightly sealed glass vials.

### 3.5. Antifungal Assays

#### 3.5.1. Screening of the Marine Extracts

*C. glabrata* (ATCC2001) and *C. albicans* (SC5314) were maintained in yeast peptone dextrose (YPD) agar plates and grown at 37 °C or 30 °C, respectively. Crude extracts were dissolved in DMSO, and 5 µL was added to the wells of a 96-well plate, containing 95 µL of RPMI-1640 medium at pH 7. Extract concentrations ranged from 125 to 12.5 µg/mL (Table 1). Cellular suspensions of *C. glabrata* or *C. albicans* (3 × 10^3^ CFU/mL) were prepared from fresh cultures grown overnight on YPD agar plates, and 100 µL was added to each well. Growth in RPMI-1640 medium was recorded after 48 h, by measuring OD_600_. The growth condition without an extract/fraction but with DMSO (control condition, 2.5% DMSO) was used as the normalization condition, after background (RPMI-1640 medium) subtraction. Growth ratios below 0.5 were considered for further analyses.

#### 3.5.2. Antifungal Susceptibility Testing

The minimal inhibitory concentration (MIC) of *C. glabrata* and *C. albicans* was determined by conducting broth microdilution assays in accordance to the CLSI (Clinical Laboratory and Standards Institute) standard method (M27-A3) [74], with few modifications. Growth in RPMI-1640 medium was recorded after 24 and 48 h at 30 °C (*C. albicans*) or 37 °C (*C. glabrata*), by measuring OD_600_. The growth condition without an extract/(sub-)fraction, but with DMSO (final concentration 2.5%), was used as the normalization condition, after background (RPMI-1640 medium) subtraction. The MIC was set as the lowest extract/(sub-)fraction concentration at which there was a ≥50% decrease in growth compared to the control (cells grown in the presence of 2.5% of DMSO). At least, three independent assays were performed for each crude extract/fraction. Fluconazole (ACROS Organics) was used as a reference antifungal. The range of tested concentrations for each extract/(sub-)fraction is listed in Table S1. The minimal fungicidal concentration (MFC) was assessed by spotting 5 μL of the above cultures onto YPD agar plates. Growth was recorded after 24 or 48 h at 30 °C (*C. albicans*) or 37 °C (*C. glabrata*). The MFC corresponds to the concentration of the fraction that decreases the number of cells compared to the initial inoculum.

### 3.6. Bioassay-Guided Fractionation of the M. arbuscula Crude Extract

Sliced bodies of *M. arbuscula* (wet weight, 29.8 g; dry weight, 15.3 g) were exhaustively extracted, as previously described, to obtain 1.70 g of a crude residue. Liquid–liquid fractionation of 1.65 g of crude extract with H_2_O/CH_2_Cl_2_ (1:1 *v/v*) produced an aqueous and organic phase. The aqueous phase was extracted with *n*-butanol (200 mL) to yield 217.0 mg of the final aqueous fraction (WF) and 756.0 mg of the *n*-butanol fraction (BF), after removal of the solvents under reduced pressure. The organic phase was concentrated under reduced pressure and was further partitioned between 10% aqueous CH_3_OH (400 mL) and hexane (2 × 400 mL) to produce, after removing the solvent under reduced pressure, 672.2 mg of the hexane fraction (HF). The H_2_O content (% *v/v*) of the methanolic fraction was adjusted to 50% aqueous CH_3_OH, and the mixture was extracted with CH_2_Cl_2_ (100 mL) to afford, after removing the solvent under reduced pressure, 106.3 mg of the CH_2_Cl_2_ fraction (DF) and 755.8 mg of the remaining aqueous methanolic fraction (WMF). The dichloromethane fraction (DF) was subjected to solid phase extraction (SPE) with RP-18 (Merck KGaA), using a stepped gradient from H_2_O to CH_3_OH and then CH_2_Cl_2_ (H_2_O (100%), H_2_O/CH_3_OH (2:1, 1:1, and 1:2), CH_3_OH (100%), CH_3_OH/CH_2_Cl_2_ (1:1), and CH_2_Cl_2_ (100%), yielding seven fractions (R1–R7). The fractions were concentrated under reduced pressure, producing the following weights: R1: 3.8 mg, R2: 1.4 mg, R3: 22.3 mg, R4: 17.7 mg, R5: 39.8 mg, R6: 17.5 mg and R7: 3.8 mg. Fractions R2–R5 were subjected to UHPLC/HRMS analysis and the mobile phase consisted of the following compounds: (A) H_2_O with 0.1% formic acid (*v/v*); (B) CH_3_CN with 0.1% formic acid (*v/v*) at a flow rate of 400 µL/min. A combination of gradient and isocratic elution was used, starting with 99% A and 1% B, changing to 1% of A and 99% of B in 13 min, followed by 2 min of isocratic at 99% of B, 1 min gradient from 99% to 1% of B and finally, 4 min of isocratic at 99% of A.

### 3.7. De-Replication

De-replication of the sub-fractions was performed by ultra high-performance liquid chromatography/high-resolution mass spectroscopy (UHPLC/HRMS) on Q Exactive Focus (Thermo Fisher Scientific, Waltham, MA, USA) coupled to UltiMate 3000 UHPLC (Thermo Fisher Scientific, Waltham, MA, USA), using Xcalibur software v.4.0.27.19 (Thermo Fisher Scientific, Waltham, MA, USA). The method consisted of several cycles of full MS scans (R = 70,000) in positive and negative modes in separate runs. External calibration was performed using the LTQ VELOS ESI Positive Ion Calibration Solution (Ref.: 11340360, Thermo Fisher Scientific, Waltham, MA, USA) and Negative Ion Calibration Solution (Ref.: 11360360, Thermo Scientific). The raw MS was analyzed using Compound Discoverer software v2.1 (Thermo Fisher Scientific, Waltham, MA, USA). The separation was performed using a Waters XBridge column C18, 2.1 × 150 mm, 3.5 µm particle size, P/N 186003023 (Optima™ LC/MS Grade, Thermo Fisher Scientific, Waltham, MA, USA). The column temperature was maintained at 30 °C and the mobile phase consisted of the following compounds: (A) H_2_O with 0.1% formic acid (*v/v*) and (B) CH_3_CN with 0.1% formic acid (*v/v*). The mass spectrometer operated in the positive ESI mode. The exact mass of the components was compared against the Antimarin^®^ database and for the components with no matches in the database, the predicted molecular formula and exact mass were searched in the database platform SciFinder^®^. If a plausible match was found, considering the exact mass/molecular formula, the molecule was considered as a putative component of the fraction. Finally, ^13^C NMR spectra were recorded on a Bruker Avance 500 spectrometer at 125 MHz, respectively, using CD_3_OD for confirming the presence of the main chemical shifts of the compounds found.

## 4. Conclusions

This work shed light on the great antifungal potential of marine natural products produced by invertebrates of the Yucatan Peninsula. Three of the nine sponge species whose extracts were active against *C. albicans* and *C. glabrata* (*H. (Rhizoniera) curacaoensis*, *A. crassa* and *A. dilatata)* have never been associated with antifungal activity, and therefore may represent a new source of antifungal compounds.

The fact that most of these extracts were more effective against *C. glabrata* is particularly interesting, as this yeast is more tolerant to the current antifungals than *C. albicans*.

*M. arbuscula* stood out as the most active species against both *C. glabrata* and *C. albicans*. This observation is in line with several reports that highlight the antifungal activity of MNPs isolated from this organism, such as batzelladine L, batzelladine D, norbatzelladine L, and ptiolomycalin A. However, by combining a bioguided fractionation with a de-replication methodology, we found that the activity of *M. arbuscula* crude extract cannot be ascribed to these compounds. Interestingly, in one of the most active sub-fractions, we found several compounds, of which we identified two—mirabilin B and penaresidin B. Mirabilin B stands out as a promising drug candidate because the pure compound is active against another yeast species—*C. neoformans*—and its synthesis has already been reported. In the future, it would be interesting to further explore the antifungal and antibiofilm properties of mirabilin B on *Candida* spp. This would be particularly important given the fungicidal activity of the sub-fraction where mirabilin B was found, which makes the future isolation and identification of the molecules responsible for that activity a possible new strategy to combat life-threatening fungal infections that affect immunocompromised individuals.

## Figures and Tables

**Table 1:** Marine crude extracts tested for antifungal activity.

Code	Species	Yield (g)	Extract Concentration (mg/mL)	Concentration Used in the Screening (µg/mL)
E38	*Aaptos* sp.	4.9	1.66	41.5
CZE56	*Agelas citrina*	1.9	5	125
E27-2	*Agelas clathrodes*	11.2	1.66	41.5
MA18-10	*Agelas clathrodes*	7.2	2.5	62.5
E25-1	*Agelas dilatata*	21.3	1.66	41.5
E26-2	*Agelas sceptrum*	4.6	2.5	62.5
E50	*Aiolochroia crassa*	5.2	5	125
MA18-4	*Aiolochroia crassa*	8.7	1.43	35.75
E29	*Amphimedon compressa*	12.9	1.66	41.5
E36	*Aplysina cauliformis*	6.3	2.5	62.5
E46	*Aplysina fistularis*	2.7	2.5	62.5
E42	*Aplysina fulva*	1.8	5	125
EY18-5	*Aplysina fulva*	2.9	1	25
E47	*Aplysina muricyanna*	4.4	2.5	62.5
BA-3	*Briareum asbestinum*	3.9	5	125
E28	*Callyspongia longissima*	1.8	1.66	41.5
E31	*Callyspongia plicifera*	1.2	1	25
E16	*Callyspongia vaginalis*	0.9	1.66	41.5
MA18-6	*Chondrilla caribensis f. hermatypica*	2.1	2.5	62.5
RIO18-1	*Chondrilla* sp.	4.6	2.5	62.5
MA18-2	*Cinachyrella kuekenthali*	2.1	0.5	12.5
EY18-11	*Clathria gomezae*	1.8	5	125
E7-E34	*Clathria virgultosa*	5.5	1	25
EY18-10	*Clathrina* sp.	1.4	2	50
T18-M1	*Clavelina* sp.	5.0	1	25
EY18-1	*Cliona delitrix*	5.2	2	50
EY18-3	*Cliona varians*	1.8	1.66	41.5
E8-2	*Didemnum perlucidum*	1.8	2.5	62.5
E01	*Didemnum* sp.	3.7	2.5	62.5
T18-M4	*Didemnum* sp.	3.5	2.5	62.5
EY18-12	*Dysidea* sp.	3.3	5	125
T18-M2	*Ecteinascidia* sp.	9.0	2.5	62.5
MA18-9	*Ectyoplasia ferox*	5.9	1.66	41.5
MA18-13	*Ectyoplasia* sp.	2.2	5	125
RIO18-T1	*Eudistoma amanitum*	3.6	2.5	62.5
TY18-2	*Eudistoma* sp.	2.9	5	125
E18-M1	*Halichondria melanadocia*	14.1	1	25
EY18-4	*Haliclona (Rhizoniera) curacaoensis*	7.9	5	125
E9-2	*Ircinia felix*	43.5	1.66	41.5
MA18-11	*Ircinia felix*	1.7	2.5	62.5
E24-2	*Ircinia strobilina*	14.1	5	125
E52	*Ircinia strobilina*	4.9	2.5	62.5
E2-2	*Leucetta floridana*	1.3	1.25	31.25
E4	*Melophlus hajdui*	4.4	2.5	62.5
T18-M6	*Molgula* sp.	3.9	5	125
E35	*Monanchora arbuscula*	29.8	2.5	62.5
MA18-1	*Mycale laevis*	14.1	1	25
MA18-5	*Mycale laevis*	4.9	5	125
CZE18	*Myrmekioderma gyroderma*	7.5	2.5	62.5
E15	*Niphates digitalis*	2.5	1	25
E49	*Niphates erecta*	1.6	1	25
MA18-12	*Niphates erecta*	5.5	5	125
MA18-7	*Niphates erecta*	2.8	2.5	62.5
TY18-1	*Phallusia nigra*	5.5	2.5	62.5
E3	*Plakinastrella onkodes*	4.9	5	125
E41	*Polycarpa* sp.	2.4	2	50
T18-M5	*Polyclinum* sp.	1.8	5	125
EY18-8	*Polysyncraton* sp.	3.6	2.5	62.5
DNY	*Scopalina ruetzleri*	29.8	1.66	41.5
E53	*Scopalina ruetzleri*	1.8	5	125
EY18-7	*Scopalina ruetzleri*	5.5	1.25	31.25
E11-2	*Spongia tubulifera*	29.8	2.5	62.5
E20	*Tethya* sp.	29.8	5	125
E7-2	*Trididemnum solidum*	3.4	2.5	62.5
EP	*Xestospongia muta*	14.1	1	25

**Table 2:** MIC values (µg/mL) of the organic extracts of marine organisms from the Yucatán Peninsula for *C. albicans* and *C. glabrata*.

		MIC (µg/mL)			
Code/ Reference	Species	*C. glabrata*		*C. albicans*	
		24 h	48 h	24 h	48 h
MA18-4	*A. crassa*	4.47	17.88	35.78	35.78
E29	*A. compressa*	2.59	2.59	5.19	5.19
E35	*M. arbuscula*	3.91	3.91	3.91	3.91
CZE56	*A. citrina*	7.81	7.81	3.91	3.91
Fluconazole	*-*	8	16	0.5	0.5

**Table 3:** Fractions obtained from the crude extract of *M. arbuscula* (E35).

Fraction	Concentration (mg/mL)	Concentration Used in the Bioassay (µg/mL)
E35-WF	4.8	120
E35-DF	2.4	60
E35-HF	2.5	62.5
E35-BF	4.7	117.5
E35-WMF	5.2	130

**Table 4:** Sub-fractions obtained from the fraction E35-DF.

Sub-Fraction	Concentration (mg/mL)	Concentration Used in the Bioassay (µg/mL)
R1	5.4	135
R2	5.2	130
R3	3.6	90
R4	2.4	60
R5	4.6	115
R6	5.4	135
R7	5.4	135

**Table 5:** MIC values (µg/mL) of fraction E35-DF and of the resulting sub-fractions R2 to R5 for *C. albicans* and *C. glabrata*.

	MIC (µg/mL)			
(Sub-)Fraction/ Reference	*C. glabrata*		*C. albicans*	
	24 h	48 h	24 h	48 h
E35-DF	3.75	3.75	7.50	15
R2	2.03	4.06	2.03	4.06
R3	2.81	5.63	2.81	5.63
R4	3.75	3.75	7.50	15
R5	1.08	3.59	1.80	3.59
Fluconazole	8	16	0.5	0.5

**Table 6:** MIC values (µg/mL) of the sub-fractions R2 to R5 for *C. krusei*, *C. tropicalis* and *C. parapsilosis*.

	MIC (µg/mL)					
Sub-Fraction	*C. krusei*		*C. tropicalis*		*C. parapsilosis*	
	24 h	48 h	24 h	48 h	24 h	48 h
R2	14.69	29.38	14.69	14.69	14.69	29.38
R3	31.25	125	15.63	31.25	15.63	15.63
R4	11.25	11.25	5.63	11.25	5.63	5.63
R5	7.19	28.75	3.59	7.19	3.59	7.19

**Table 7:** MFC values (µg/mL) of the sub-fractions R2 to R5.

	MFC (µg/mL)							
	24 h				48 h			
	R2	R3	R4	R5	R2	R3	R4	R5
*C. albicans*	14.69	31.25	11.25	14.38	*	31.25	5.63	7.19
*C. glabrata*	58.75	125.00	11.25	3.59	58.75	125.00	22.50	3.59
*C. krusei*	117.5	*	22.5	57.50	58.75	*	22.5	57.50
*C. tropicalis*	14.69	15.63	11.25	7.19	14.69	15.63	11.25	7.19
*C. parapsilosis*	29.38	7.81	11.25	7.19	29.38	31.25	45.00	57.50

* No fungicidal activity detected within the concentration range tested.

**Table 8:** [M + H]^+^ ion adducts detected in UHPLC-HRMS analyses of R2, R3, R4 and R5 sub-fractions.

Sub-Fractions	UHPLC (Retention Time in min)	[M + H]^+^ ion Adducts(*m/z*)	Possible Compound *
R2	9.56	256.8662	-
	10.49	259.2150	-
	15.49	282.2792	-
R3	12.00	330.3001	Penaresidin B (*m/z* 330.3002) isolated from *Penares* sp. [31,32]
	12.21	404.3271	-
R4	9.12	246.1965	Mirabilin B (*m/z* 246.1964) isolated from *Arennochalina mirabilis* [33]
	9.98	248.2177	-
	10.25	346.2488	-
	10.75	348.2644	-
	11.00	376.2960	-
	11.25	318.3001	-
	11.00	330.3001	Penaresidin B (*m/z* 330.3002) isolated from *Penares* sp. [31,32]
R5	10.87	325.2749	-
	11.22	318.3006	-
	11.71	332.3156	-
	11.90	330.3001	Penaresidin B (*m/z* 330.3002) isolated from *Penares* sp. [31,32]
	12.14	404.3265	-
	12.42	344.3158	-
	15.49	282.2793	-

* The positive criterion corresponds to an *m/z*-accuracy of 3 decimal places.

**Table 9:** Experimental^a^ and reported^b 13^C NMR (125 MHz) data for mirabilin B (**1**) and penaresidin B (**2**).

Post.	1		2	
	*^a^δ*_C_, Type *	*^b^δ*_C_, Type **	*^a^δ*_C_, Type *	*^b^δ*_C_, Type **
1			61.3, CH_2_	62.3, CH_2_
2	176.3, C	176.2, C	66.3, CH	66.6, CH
3	126.9, C	126.8, C	66.9, CH	67.4, CH
4	167.3, C	167.3, C	65.4, CH	64.8, CH
5			27.2, CH_2_	26.9, CH_2_
6	164.6, C	164.6, C	27.2, CH_2_	26.9, CH_2_
7			27.2, CH_2_	26.9, CH_2_
8	39.2, CH	39.0, CH	27.2, CH_2_	26.9, CH_2_
9	40.4, CH_2_	40.8, CH_2_	27.2, CH_2_	26.9, CH_2_
10	35.1, CH	35.2, CH	27.2, CH_2_	26.9, CH_2_
11	48.8, CH	48.2, CH	27.2, CH_2_	26.9, CH_2_
12	34.4, CH_2_	34.3, CH_2_	27.2, CH_2_	26.9, CH_2_
13	34.2, CH_2_	34.1, CH_2_	27.2, CH_2_	26.9, CH_2_
14			72.6, CH	72.7, CH
15	21.6, CH_3_	21.3, CH_3_	34.9, CH_2_	34.9, CH_2_
16	31.6, CH_3_	31.1, CH_3_	24.8, CH	24.6, CH
17	28.6, CH_2_	28.6, CH_2_	25.6, CH_2_	25.5, CH_2_
18	24.2, CH_2_	24.4, CH_2_	11.5, CH_3_	11.6, CH_3_
19	14.4, CH_3_	14.4, CH_3_	22.3, CH_3_	22.3, CH_3_

* NMR experiment run in CDCl_3_ solvent; ** NMR experiment run in CD_3_OD solvent.

**Table 10:** Taxonomic information and previously reported antifungal activity of the marine species studied in this work.

Order	Family	Species (Code Used in This Study)	Antifungal Activity Previously Reported	Reference
Aplousobranchia	Clavelinidae	*Clavelina* sp. (T18-M1)	(2S,3R)-2-aminododecan-3-ol isolated from *Clavelina oblonga* active against *C. albicans* and *C. glabrata*. Indolizines isolated from *C. picta* active against *C. albicans*.	[37,38]
	Didemnidae	*Didemnum perlucidum* (E8-2)	No	
		*Didemnum* sp. (T18-M4) (E01)	Didemnoline B and C active on *S. cerevisiae*. Lepadin D and E active against *Ustilago violacea* (now *Microbotryum violaceum)* and *Eurotium repens*, respectively. Lepadin F active against *E. repens*. Didemnaketal F and G active against *C. albicans.* β-carboline active against *C. albicans*, *C. intermedia* and *C. krusei.* β-carboline dimer active against *C. intermedia*. β-carboline N-Me salts showed activity against *C. intermedia* and *C. krusei*.	[38,39,40,41]
		*Trididemnum solidum* (E7-2)	No	
	Polycitoridae	*Polysyncraton* sp. (EY18-8)	No	
		*Eudistoma amanitum* (RIO18-T1)	No	
		*Eudistoma* sp. (TY18-2)	Eudistomin W and X active against *C. albicans*.	[27]
	Polyclinidae	*Polyclinum* sp. (T18-M5)	No	
Phlebobranchia	Ascidiidae	*Phallusia nigra* (TY18-1)	No	
	Perophoridae	*Ecteinascidia* sp. (T18-M2)	No	
Stolidobranchia	Molgulidae	*Molgula* sp. (T18-M6)	No	
	Styelidae	*Polycarpa* sp. (E41)	No	
Alcyonacea	Briareridae	*Briareum asbestinum* (BA-3)	No	
Agelasida	Agelisidae	*Agelas citrina* (CZE56)	(–)-Agelasidine C, agelasidine E and F active against *C. albicans*.	[21,22]
		*Agelas clathrodes* (E27-2) (MA18-10)	Clathramides A and B showed activity against *Aspergillus niger*.	[42]
		*Agelas dilatata* (E25-1)	No	
		*Agelas sceptrum* (E26-2)	Sceptrin is active against *C. albicans*, *Alternaria* sp. and *Cladosporium cucumerinum*.	[25]
*Axinelida*	Heteroxyidae	*Myrmekioderma gyroderma* (CZE18)	No	
	Raspailiidae	*Ectyoplasia ferox* (MA18-9)	No	
		*Ectyoplasia* sp. (MA18-13)	No	
Chondrilida	Chondrilidae	*Chondrilla caribensis f. hermatypica* (MA18-6)	No	
		*Chondrilla* sp. (RIO18-1)	No	
Clathrinida	Clathrinidae	*Clathrina* sp. (EY18-10)	Clathridine is active against *C. albicans* and *S. cerevisiae*.	[43]
	Leucittidae	*Leucetta floridana* (E2-2)	Extract showed activity against *C. albicans*.	[26]
Clionaida	Clionaidae	*Cliona delitrix* (EY18-1)	No	
		*Cliona varians* (EY18-3)	No	
Dictyoceratida	Dysideidae	*Dysidea* sp. (EY18-12)	3′-hydroxyavarone, 3′,6′di-hydroxyavarone and 6′-acetoxyavarol are active against *C. albicans*. 9α,11α-epoxycholest-7-ene-3β,5α,6α,19-tetrol 6-acetate (ECTA) is active against *C. albicans.* 3,5-dibromo-2-(3,5-dibromo-2-methoxyphenoxy) phenol is active against *C. albicans*, *C. glabrata*, *C. tropicalis*, *A. fumigatus*, *A. flavus* and *A. niger*. Puupehenone is active against *C. albicans*. Synthetic (*Z*)-dysidazirine [(-)-1] is active against *C. albicans*, *C. glabrata* and *C. krusei.* Sesterterpenes sulphates showed inhibitory activity against *C. albicans*. Avarol is active against eight *Candida* spp.	[44,45,46,47,48,49,50,51,52,53]
	Irciniidae	*Ircinia felix* (E9-2) (MA18-11)	Extract showed activity against *C. tropicalis*.	[54]
		*Ircinia strobilina* (E24-2) (E52)	No	
	Spongiidae	*Spongia tubulifera* (E11-2)	No	
Haplosclerida	Callyspongiidae	*Callyspongia longissima* (E28)	No	
		*Callyspongia plicifera* (E31)	No	
		*Callyspongia vaginalis* (E16)	No	
	Chalinidae	*Haliclona (Rhizoniera) curacaoensis* (EY18-4)	No	
	Niphatidae	*Amphimedon compressa* (E29)	8,8′-dienecyclostellettamine is active against *C. albicans* and *A. fumigatus.*	[23,24]
		*Niphates digitalis* (E15)	No	
		*Niphates erecta* (E49) (MA18-7) (MA18-12)	No	
	Petrosiidae	*Xestospongia muta* (EP)	Xestospongiamide is active against *A. niger* and *C. albicans*. Xestospongin C and D are active against fluconazole-resistant *Candida* spp.	[55,56,57]
Homosclerophorida	Plakinidae	*Plakinastrella onkodes* (E3)	Plakinic acid F is active against *C. albicans* and *A. fumigatus*. Epiplakinic acid F is active against *C. albicans* and *A. fumigatus*. 1,2-dioxane ring peroxide acid is active against *C. albicans* and *A. fumigatus*. Plakortide F is active against *C. albicans*. 1,2-dioxolane perocide acid is active against *C. albicans*.	[58,59,60,61]
Poecilosclerida	Crambeidae	*Monanchora arbuscula* (E35)	Dehydrobatzelladine C is active against *C. albicans* and *A. fumigatus*. Batzelladine L shows activity against *A. flavus*. Mirabilin B is active against *C. neoformans*.	[61,62,63,64]
	Microcionidae	*Clathria gomezae* (EY18-11)	No	
		*Clathria (Thalysisas) virgultosa* (E7-E34)	No	
	Mycalidae	*Mycale laevis* (MA18-1) (MA18-5)	No	
Scopalinida	Scopalinidae	*Scopalina ruetzleri* (DNY) (E53) (EY18-7)	Extract showed activity against *C. albicans*.	[26]
Suberitida	Halichondriidae	*Halichondria melanadocia* (E18-M1)	No	
	Subertidae	*Aaptos* sp. (E38)	3-(phenethylamino)demethyl(oxy)aaptamine is active against *C. albicans*, *C. parapsilosis*, *Trichophyton rubrum* and *Microsporum gypseum*.	[65,66,67]
Tethyida	Tethyidae	*Tethya* sp. (E20)	Extract from species of this genus showed mild activity against *C. albicans*.	[68,69]
Tetractinellida	Geodiidae	*Melophlus hajdui* (E4)	No	
	Tetillidae	*Cinachyrella kuekenthali* (MA18-2)	No	
Verongiida	Aplysinidae	*Aiolochroia crassa* (E50) (MA18-4)	No	
		*Aplysina cauliformis* (E36)	No	
		*Aplysina fistularis* (E46)	Lovastatin is active against *Candida*, *Aspergillus*, *Fusarium* and *Trichophyton* species.	[70]
		*Aplysina fulva* (E42) (EY18-5)	Lectin is active against *C. albicans* and *C. tropicalis*.	[71,72,73]
		*Aplysina muricyana* (E47)	No	

## Data Availability

Not applicable.

## Supplementary Materials

The following supporting information can be downloaded at: https://www.mdpi.com/article/10.3390/molecules28020606/s1, Figure S1: UHPLC chromatogram of R2 fractions from UHPLC-HRMS experiment. Figure S2: UHPLC chromatogram of R3 fractions from UHPLC-HRMS experiment. Figure S3: UHPLC chromatogram of R5 fractions from UHPLC-HRMS experiment. Figure S4: ^13^C NMR spectrum of R4 subfraction with the main chemical shifts of 1. Figure S5: HRMS-ESI of the compound 1. Figure S6: ^13^C NMR spectrum of R4 subfraction with the main chemical shifts of 2. Figure S7: HRMS-ESI of the compound 2. Table S1: Concentration range tested for MIC and MFC determination.
