# Supplementary material for: Antifungal Potential of Marine Organisms of the Yucatan Peninsula (Mexico) against Medically Important Candida spp
Source: Molecules. 2023 Jan 6;28(2):606. doi: 10.3390/molecules28020606 (PMC9863849; doi:10.3390/molecules28020606)
Supplement: Supplementary file 1 [file molecules-28-00606-s001.zip › molecules-2053080-supplementary.pdf]

Supplementary Materials

Antifungal Potential of Marine Organisms of the Yucatan Peninsula (Mexico) against Medically Important *Candida* spp.

Dawrin Pech-Puch [1,2], Diana Grilo [3], Susana Eunice Calva-Pérez [1], Andreia Pedras [3], Harold Villegas-Hernández [1], Sergio Guillén-Hernández [1], Raúl Díaz-Gamboa [1], Mateo Forero Tunjano [4], Jaime Rodríguez [4], Oscar A. Lenis-Rojas [3,*], Carlos Jiménez [4,*] and Catarina Pimentel [3,*]

[1] Departamento de Biología Marina, Universidad Autónoma de Yucatán, Km. 15.5, Carretera Mérida-Xmatkuil, A.P. 4-116 Itzimná, Merida C.P. 97100, Mexico

[2] Escuela Nacional de Estudios Superiores Unidad Mérida (ENES Mérida), Universidad Nacional Autónoma de México (UNAM), Carretera Mérida-Tetiz km 4.5, Tablaje, Catastral No. 6998, Municipio de Ucú, Ucú C.P. 97357, Mexico

[3] Instituto de Tecnologia Química e Biológica António Xavier (ITQB NOVA), Universidade Nova de Lisboa, Oeiras, 1070-312 Lisbon, Portugal

[4] Centro de Interdisciplinar de Química e Bioloxía (CICA), Facultade de Ciencias, Universidade da Coruña, 15071 Coruña, Spain

* Correspondence: oscar.rojas@itqb.unl.pt (O.A.L.-R.); carlos.jimenez@udc.es (C.J.); pimentel@itqb.unl.pt (C.P.)

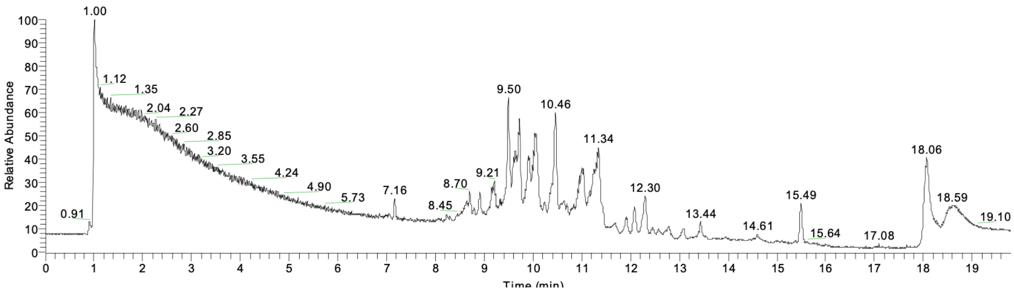

Figure S1. UHPLC chromatogram of R2 fraction from UHPLC-HRMS experiment.

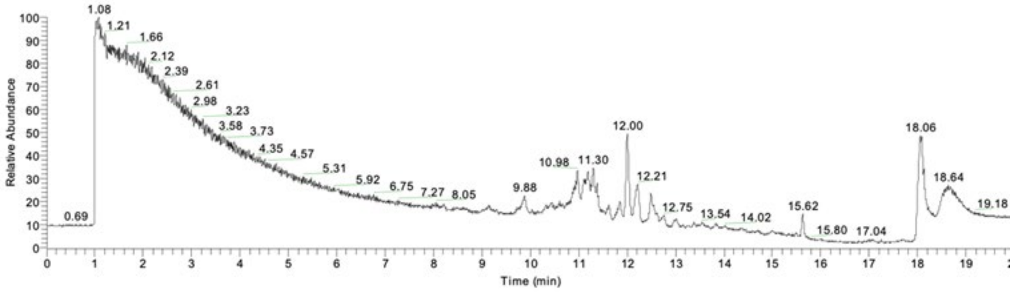

Figure S2. UHPLC chromatogram of R3 fraction from UHPLC-HRMS experiment.

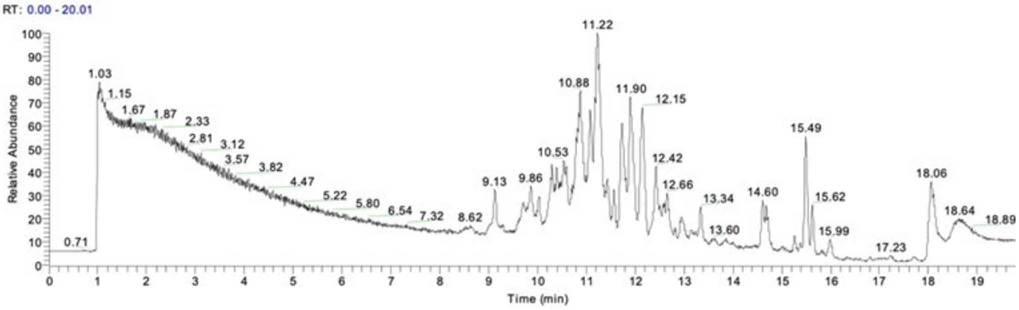

Figure S3. UHPLC chromatogram of R5 fraction from UHPLC-HRMS experiment.

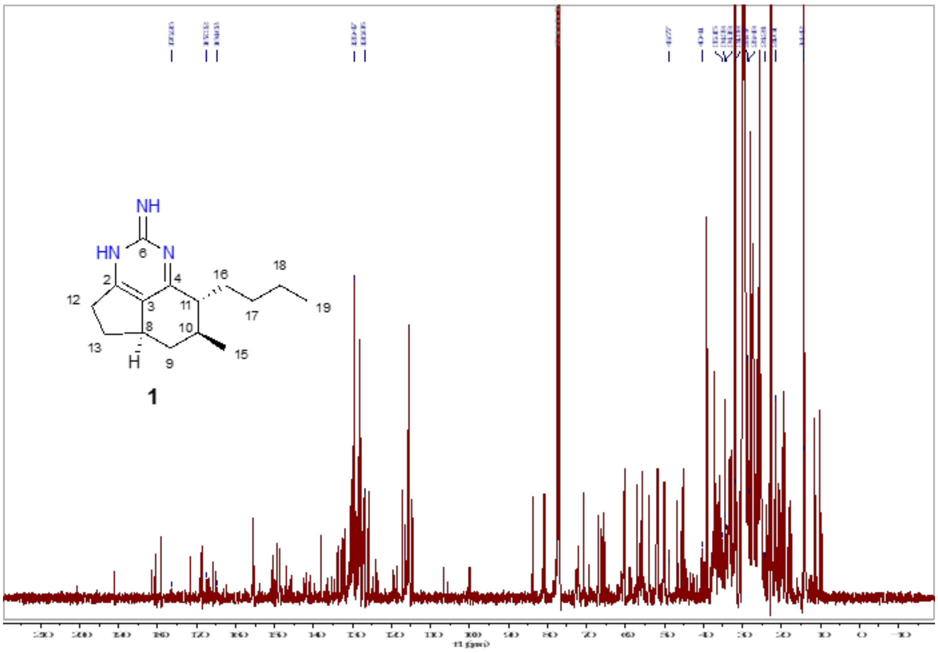

Figure S4. ^{13}C NMR spectrum of R4 subfraction with the main chemical shifts of **1.**

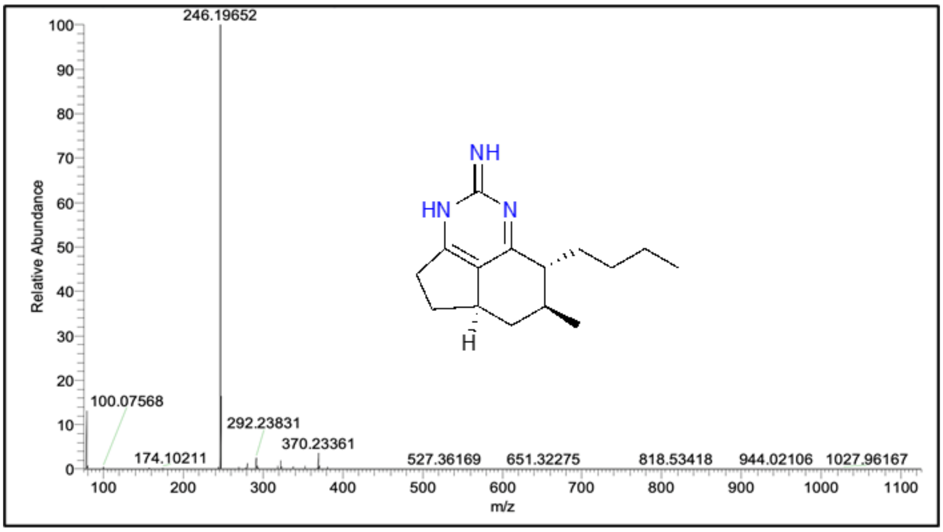

Figure S5. HRMS-ESI of the compound **1.**

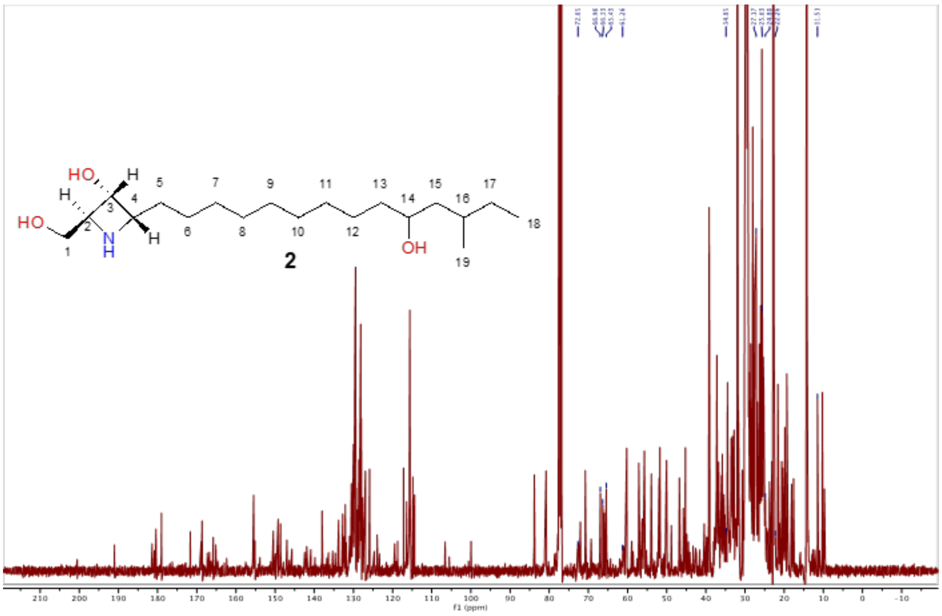

Figure S6. ^{13}C NMR spectrum of R4 subfraction with the main chemical shifts of **2.**

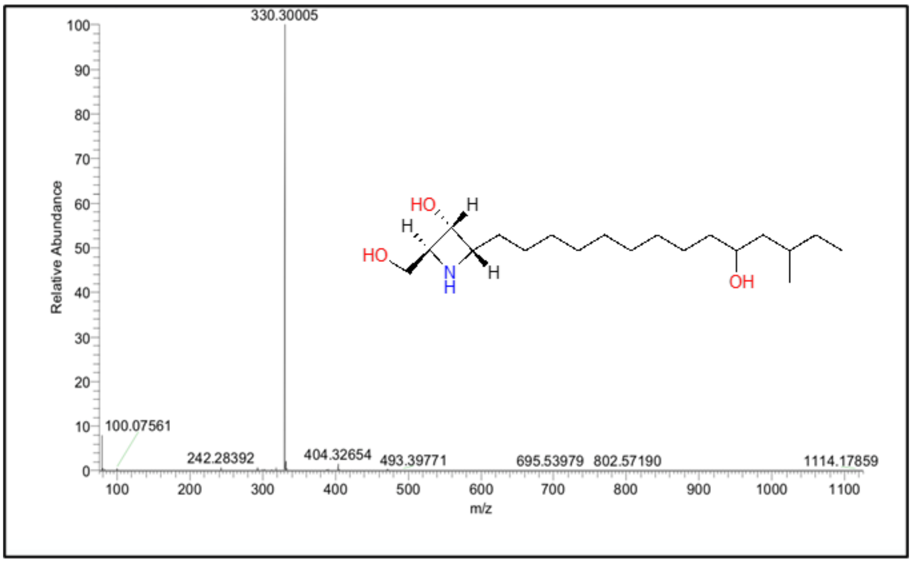

Figure S7. HRMS-ESI of the compound **2.**

Table S1. Concentration range tested for MIC and MFC determination.

	**Concentration range tested (µg/mL)**
Crude extract	
CZE56	125–0.244
MA18-4	35.75–0.070
E29	41.5–0.081
E35	62.5–0.122
Sub-fractions of E35	
DF	60.00–0.117
BF	117.50–0.229
WMF	130.00–0.254
Sub-Fraction	
R1	135–0.264
R2	130–0.254
R3	80–0.176
R4	60–0.117
R5	115–0.225
R6	135–0.264
R7	135–0.264
